# Complete Annotated Genome Assembly of Flax Pathogen *Colletotrichum lini*

**DOI:** 10.3390/jof10090605

**Published:** 2024-08-26

**Authors:** Elizaveta A. Sigova, Ekaterina M. Dvorianinova, Tatiana A. Rozhmina, Ludmila P. Kudryavtseva, Daiana A. Zhernova, Antoniy M. Kaplun, Valeria A. Pavlova, Yakov V. Bodrov, Alexander A. Arkhipov, Elena V. Borkhert, Elena N. Pushkova, Nataliya V. Melnikova, Alexey A. Dmitriev

**Affiliations:** 1 Engelhardt Institute of Molecular Biology, Russian Academy of Sciences, Moscow 119991, Russia; dvorianinova.em@phystech.edu (E.M.D.); zhernova.d@yandex.ru (D.A.Z.); kaplun.am@phystech.edu (A.M.K.); valeria_pavl.1@mail.ru (V.A.P.); yakov.bodrov@list.ru (Y.V.B.); arkhipov.aleksandr2.0@gmail.com (A.A.A.); sashai@inbox.ru (E.V.B.); pushkova18@gmail.com (E.N.P.); mnv-4529264@yandex.ru (N.V.M.); 2 Federal Research Center for Bast Fiber Crops, Torzhok 172002, Russia; tatyana_rozhmina@mail.ru (T.A.R.); lpkudryavtseva@icloud.com (L.P.K.); 3 Faculty of Biology, Lomonosov Moscow State University, Moscow 119234, Russia; 4 Moscow Institute of Physics and Technology, Moscow 141701, Russia; 5 Lomonosov Institute of Fine Chemical Technologies, MIREA—Russian Technological University, Moscow 119571, Russia; 6 I.M. Sechenov First Moscow State Medical University, Moscow 119991, Russia

**Keywords:** *Colletotrichum lini*, anthracnose, flax pathogen, complete genome assembly, genome annotation, effector proteins

## Abstract

*Colletotrichum lini* is a fungal pathogen of flax that can cause significant yield and quality losses. In this work, we obtained the first complete annotated genome assembly of the highly virulent *C. lini* strain #394-2. The nuclear genome consisted of ten core and two accessory chromosomes and had a length of 53.7 Mb. The mitochondrial genome was 39.1 kb. The assembly was obtained by the Canu–Racon ×2–Medaka–Polca algorithm using Oxford Nanopore Technologies and Illumina data. As a result of the annotation with the Illumina RNA-Seq data, 12,449 genes were identified. Potential signaling proteins were tested for effector functions and 550 effector proteins were predicted using EffectorP. The visualization of the effector protein localization revealed that the presence of effector proteins was associated with repeat-rich regions. A comparison of the genomic structure of *C. lini* with chromosome-level and complete assemblies of the genus *Colletotrichum* representatives revealed that the genomes of *Colletotrichum* species differed by the presence of chromosomal rearrangements. The obtained assembly expands the knowledge of the genomic structure of *Colletotrichum* species and provides the basis for further studies of *C. lini*, which will help to understand the virulence mechanisms and protect flax from anthracnose.

## 1. Introduction

*Colletotrichum lini* is the anthracnose causative agent of flax. This pathogen leads to significant crop losses, destroying young sprouts and mature plants [1,2,3,4]. Thus, anthracnose causes serious damage to the economies of the countries where flax is an agriculturally valuable crop [5,6]. Flax oil and fiber are used in many industries [7,8,9]. Therefore, saving and increasing flax yields is critically important for many uses of flax products.

Currently, the complete genomic organization of *C. lini* is unknown, since there is no available chromosome-level assembly [10,11]. Despite the fact that the NCBI database already contains rather continuous assemblies of the phytopathogen genome (https://www.ncbi.nlm.nih.gov/datasets/genome/?taxon=500171, accessed on 1 July 2024), the lack of a chromosome-level assembly inhibits further quality genomic and transcriptomic studies on *C. lini*. Currently, about 250 species are recognized in the genus *Colletotrichum* [12], and only seven complete or chromosome-level assemblies for members of the genus are available in the NCBI database (https://www.ncbi.nlm.nih.gov/datasets/genome/?taxon=5455&assembly_level=2:3, accessed on 1 July 2024).

*Colletotrichum* species representatives vary at the genomic level and infect different hosts. The study of their genomes suggested a relation between the degree of pathogenicity and the species organization at the molecular level. Supposedly, the virulence of anthracnose-causing strains is associated with accessory chromosomes (mini-chromosomes) [13]. These chromosomes are involved in horizontal gene transfer [14] and could have appeared during the recombination of the main chromosomes. However, in the absence of chromosome-level genome assembly, studying and comparing such chromosomes with the conserved ones is difficult. The chromosome-level assembly of the *Colletotrichum graminicola* genome sheds light on the characteristics of core and accessory chromosomes [15]. However, virulence genes remain unknown.

For breeding resistant flax varieties and developing anthracnose control techniques, the genes responsible for pathogenicity should be revealed, as well as their location in the genome. Gaining this knowledge is impossible without obtaining a chromosome-level assembly. When studying species *C. fructicola*, *C. siamense*, *C. aenigma*, *C. tropicale*, and *C. viniferum*, the location of the discovered effector protein genes was identified. Some of these proteins were presumably associated with the virulence level, and their genes were associated with genome repeat-rich regions [16]. The genes and mechanisms responsible for pathogenicity and infection in host plants may vary from species to species. Therefore, it is necessary to study the *C. lini* genome individually. The chromosome-level assembly should allow for determining the location of the key virulence genes and establishing the mechanisms of *C. lini* pathogenicity. In addition, since the products of virulence genes can trigger host defense, plant resistance mechanisms can also be understood more deeply.

In this work, we obtained a complete assembly of the *C. lini* genome using Oxford Nanopore Technologies and Illumina data and annotated it with Illumina RNA-Seq data. The identified genes and effector proteins were analyzed, as well as their distribution across the genome.

## 2. Materials and Methods

### 2.1. Fungal Material

The highly virulent *C. lini* strain #394-2 was provided by the Institute for Flax (Torzhok, Russia). Mycelium was provided in tubes with potato dextrose agar.

The virulence level was studied with two tests. In the first test, two varieties of flax were used—resistant Leona and susceptible Punjab. The second test was performed on twelve different flax varieties: 130-3, 130-3 × 138, 130-3 × Crystal, 130-3 × (Leona × Aoyagi), C-255, Crystal × C-255, Punjab, Leona × Crystal, C-255 × 130-3, 138 × Crystal, C-255 × A-93, and Entre-Rios. During the tests, 10–25 seeds were sown per pot for each variety. Three biological replicates were used. Plants were infected by spraying a suspension of spores of the pathogen (150–300 spores per cm^3^). The inoculated plants were covered with polyethylene isolators and kept in humid chamber conditions for 48 h. The development of anthracnose was assessed on the eighth and ninth day. The average percent of the dead infected plants from both experiments was calculated. The degree of pathogenicity was assigned according to the introduced scale: 0–30%—low pathogenicity, 31–50%—moderate pathogenicity, and 51–100%—high pathogenicity. The obtained value of 69.6% was classified as a high degree of pathogenicity.

### 2.2. DNA Extraction and Purification

For DNA extraction, we used our previously developed protocol [17] with some modifications: the mycelium was grown in a petri dish in a liquid potato dextrose medium with kanamycin (0.2 mg/mL). Then, a flask with potato dextrose medium was inoculated with the grown material and incubated on a shaker at 20–22 °C for 2–3 days. For DNA extraction, 1 g of mycelium was filtered from liquid culture through a strainer (Corning, Corning, NY, USA). Incubation time with proteinase K (>600 mAU/mL; Qiagen, Chatsworth, CA, USA) was increased from 40 min to 90 min. The obtained DNA was used for the Oxford Nanopore Technologies (ONT) and Illumina library preparation. The evaluation of the quality and quantity of the extracted DNA was performed with spectrophotometry (NanoDrop 2000C; Thermo Fisher Scientific, Waltham, MA, USA), fluorometry (Qubit 4.0; Thermo Fisher Scientific, Waltham, MA, USA), and agarose gel electrophoresis (2% agarose) techniques. For the library preparation, the obtained DNA with A260/280 = 1.9, A260/230 = 2.2, and a concentration of 430 ng/μL was used.

### 2.3. RNA Extraction and Purification

For RNA extraction, we used our previously developed CTAB-based protocol [18]. Frozen mycelium from a single batch was used for the RNA and DNA extraction. The mycelium was fragmented in a 2 mL tube, transferred into a tube with 1 mL of CTAB buffer preheated to 65 °C, and incubated at 65 °C for 30 min. The concentration and quality of the obtained RNA were assessed using the Qubit 4.0 fluorometer (Thermo Fisher Scientific, Waltham, MA, USA) and agarose gel electrophoresis (2% agarose), respectively.

### 2.4. DNA Library Preparation and Sequencing on the Oxford Nanopore Technologies and Illumina Platforms

For sequencing on the ONT platform, the SQK-LSK114 Ligation Sequencing Kit (ONT, Oxford, UK) was used for library preparation. Sequencing was performed on a PromethION instrument with an R10.4.1 flow cell (ONT, Oxford, UK).

The Illumina library was prepared using a NEBNext Ultra II DNA Library Prep Kit for Illumina (New England Biolabs, Ipswich, MA, USA) according to the manufacturer’s protocol and sequenced on a NovaSeq 6000 (Illumina, San Diego, CA, USA) instrument (150 + 150 bp).

### 2.5. cDNA Library Preparation and Sequencing on the Illumina Platform

The preparation of a cDNA library was carried out according to the protocol of Di et al. [19] with modifications, which were mainly related to the use of reagents from other manufacturers. To prepare the cDNA library, 700 ng of total RNA treated with DNase I (diaGene, Moscow, Russia) were used. To isolate the mRNA fraction, the NEBNext Poly(A) mRNA Magnetic Isolation Module kit (New England Biolabs, Ipswich, MA, USA) was used according to the manufacturer’s protocol. At the last step, the elution was performed in 6 µL of Tris buffer from the kit. The reverse transcription was performed using the Mint reverse transcriptase kit (Evrogen, Moscow, Russia) according to the manufacturer’s protocol. The cDNA library was prepared according to our previously developed protocol based on in-house Tn5 transposase [20] with preannealed oligonucleotides obtained according to the protocol of Picelli et al. [21]. The quality and concentration of the cDNA library were evaluated using the Qsep1-Plus capillary electrophoresis system (BiOptic, New Taipei City, Taiwan) and the Qubit 4.0 fluorometer (Thermo Fisher Scientific, Waltham, MA, USA), respectively. Sequencing of the obtained cDNA library was performed on NextSeq 2000 (Illumina, San Diego, CA, USA) using the NextSeq 2000 P3 kit (100 cycles) (Illumina, San Diego, CA, USA) with a read length of 51 + 51 nucleotides.

### 2.6. Genome Assembly and Polishing

The obtained ONT reads were basecalled using Guppy 6.0.1 and the dna_r10.4_e8.1_sup.cfg config file with the quality filtration threshold min_qscore = 10. Porechop 0.2.4 (https://github.com/rrwick/Porechop, accessed on 1 July 2024) was used for removing adapters. The obtained short Illumina reads were processed using Cutadapt 2.8 (-a AGATCGGAAGAG -A AGATCGGAAGAG) [22] and Trimmomatic 0.39 (PE, SLIDINGWINDOW:3:28, MINLEN:50) [23]. The draft *C. lini* strain #394-2 genome assembly was produced by Canu 2.2 (-nanopore-raw; -minInputCoverage = 5; -stopOnLowCoverage = 5; -genomeSize = 55m) [24].

The obtained draft assembly was polished with ONT reads with Racon 1.4.20 (two iterations) [25] and Medaka 1.5.0 (https://github.com/nanoporetech/medaka, accessed on 1 July 2024). Polca (MaSuRCA 4.1.0) [26] was used for polishing with Illumina reads. If required, all prior alignments before polishing were produced with Minimap2 [27]. The mitochondrial genome was detected in the resulting genome assembly using the previously obtained mitochondrial genome of *C. lini* and the local command-line BLAST 2.9.0+ tool (Basic Local Alignment Search Tool) [28].

To analyze the quality of the obtained assemblies, statistics of completeness, contiguity, and accuracy were calculated using BUSCO 5.3.2 (glomerellales_odb10) and QUAST 5.0.2 [29,30]. The following reference genome was used for QUAST reference-based statistics: *Colletotrichum higginsianum* IMI 349063 (NCBI Genome, GCA_001672515.1).

### 2.7. Genome Analyses

Tidk 0.2.31 was used for the identification of telomeric repeats and their visualization (https://github.com/tolkit/telomeric-identifier, accessed on 1 July 2024). RNA-Seq data were trimmed and filtered using PPLine [31] and used for genome annotation with Braker (--fungus) [32]. RepeatMasker 4.1.5 (-xsmall) was used for the identification of repeat content (https://www.repeatmasker.org/, accessed on 1 July 2024). STAR 2.5.2b was used for aligning RNA-Seq reads to the genome assembly [33].

The longest protein isoforms were determined using Agat (agat_sp_keep_longest_isoform.pl and agat_sp_extract_sequences.pl scripts) (https://www.doi.org/10.5281/zenodo.3552717, accessed on 1 July 2024). For functional annotation, InterProScan 5.65-97.0 was used (https://github.com/ebi-pf-team/interproscan, accessed on 1 July 2024). SignalP-6.0 predicted the presence of signal peptides in protein sequences received after genome annotation (https://services.healthtech.dtu.dk/services/SignalP-6.0/, accessed on 1 July 2024). The identified proteins with signal peptides were checked with EffectorP-3.0, which predicts effector properties [34]. The available chromosome-level and complete genome assemblies of *Colletotrichum* species (*Colletotrichum graminicola* (GCA_029226625.1), *Colletotrichum higginsianum* (GCA_001672515.1), *Colletotrichum destructivum* (GCA_034447905.1), *Colletotrichum fructicola* (GCA_039271565.1), *Colletotrichum gigasporum* (GCA_024584535.1), *Colletotrichum lupini* (GCA_023278565.1), and *Colletotrichum siamense* (GCA_038023885.1)) were aligned to the obtained *C. lini* strain #394-2 assembly using LAST 1471 (https://gitlab.com/mcfrith/last, accessed on 1 July 2024).

Visualization was performed with the BioCircos (https://CRAN.R-project.org/package=BioCircos, accessed on 1 July 2024), RIdeogram [35], chromoMap (https://CRAN.R-project.org/package=chromoMap, accessed on 1 July 2024), forcats, RColorBrewer (https://CRAN.R-project.org/package=RColorBrewer, accessed on 1 July 2024), dplyr (https://CRAN.R-project.org/package=dplyr, accessed on 1 July 2024), hrbrthemes (https://CRAN.R-project.org/package=hrbrthemes, accessed on 1 July 2024), and ggplot2 [36] R packages (https://www.R-project.org/, accessed on 1 July 2024).

## 3. Results

### 3.1. Genome Assembly and Polishing

**Figure 1:**
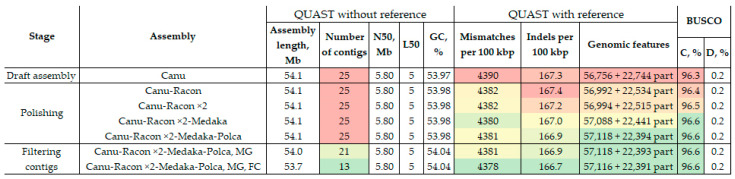
QUAST and BUSCO statistics of the *C. lini* strain #394-2 genome assembly at each step. «Filtering contigs» included 2 stages: MG—assembly with only one mitochondrial genome copy; FC—assembly with only one mitochondrial genome copy and with excluded contigs of less than 90 kb length. BUSCO: C—complete, D—duplicated. The used colors indicate estimations of the value quality: from green (best) to red (worst). Reference genome—*C. higginsianum* IMI 349063 (NCBI Genome, GCA_001672515.1).

For the genome of the highly virulent *C. lini* strain #394-2, we received 4.1 Gb of raw ONT reads with an N50 of 14.5 kb and 16 million Illumina reads (150 + 150 bp). After basecalling using Guppy with the quality filtration threshold min_qscore = 10, we obtained 2.4 Gb of basecalled ONT reads. The assembly and polishing of the *C. lini* genome were made according to our previously developed scheme—assembling long ONT reads with Canu and polishing with Racon ×2 (ONT reads)—Medaka (ONT reads)—Polca (Illumina reads) [10,37]. This algorithm has proven to be the most suitable for obtaining the most contiguous and complete fungus genome assembly. The contiguity and completeness of the obtained assemblies were analyzed at every step using QUAST with and without a reference genome and BUSCO (Figure 1, Supplementary Table S1).

As expected, the contiguity of the assembly did not improve after polishing, unlike the accuracy and completeness. The BUSCO completeness increased from 96.3% to 96.6% in the first three rounds. The last step of polishing with Polca did not change the BUSCO completeness. Most reference-based QUAST parameters were improved after every round of polishing. After all polishing rounds, mismatches per 100 kbp decreased from 4390 to 4381, indels per 100 kbp decreased from 167.3 to 166.9, and complete genomic features increased from 56,756 (and 22,744 partial) to 57,118 (and 22,394 partial). The reference genome fraction covered was improved after the first two rounds from 57.43% to 57.52%. However, after polishing with Medaka and Polca, it slightly decreased to 57.50% and 57.49%, respectively. Thus, the fully polished assembly of the *C. lini* strain #394-2 genome consisted of 25 contigs with an N50 of 5.8 Mb and a BUSCO completeness of 96.6%. Chromosome-level and complete assemblies of *Colletotrichum* genomes from the NCBI Genomes database (https://www.ncbi.nlm.nih.gov/datasets/genome/?taxon=5455&assembly_level=2:3, accessed on 1 July 2024) consist of 10-13 chromosomes and 10-25 scaffolds and have N50 values of 5.2–9.2 Mb. Thus, we obtained an assembly of quality comparable to one of the available chromosome-level and complete *Colletotrichum* assemblies.

For the further improvement of the *C. lini* strain #394-2 assembly, we searched for the mitochondrial genome. For the BLAST analysis, the *C. lini* mitochondrial genome of the previously obtained assembly of low-virulent strain #771 [10] was used. As a result of the BLAST analysis, it turned out that five contigs consisted of the mitochondrial genome in many copies. We left one full copy of the mitochondrial genome as a separate contig, the length of which was 39.1 kb. After the deletion of the repeating parts of the mitochondrial genome, the assembly comprised 21 contigs. We also analyzed the QUAST and BUSCO statistics for this assembly (Figure 1, Supplementary Table S1). The parameters presented in Figure 1 did not change except for the number of contigs, the assembly length, and the GC content. The increase in the GC percent is explained by the difference in the GC content between the nuclear and mitochondrial genomes of *C. lini*. Reference-based parameters were almost unchanged. However, the number of unaligned contigs decreased because of the deleted copies of the mitochondrial genome contigs.

**Figure 2:**
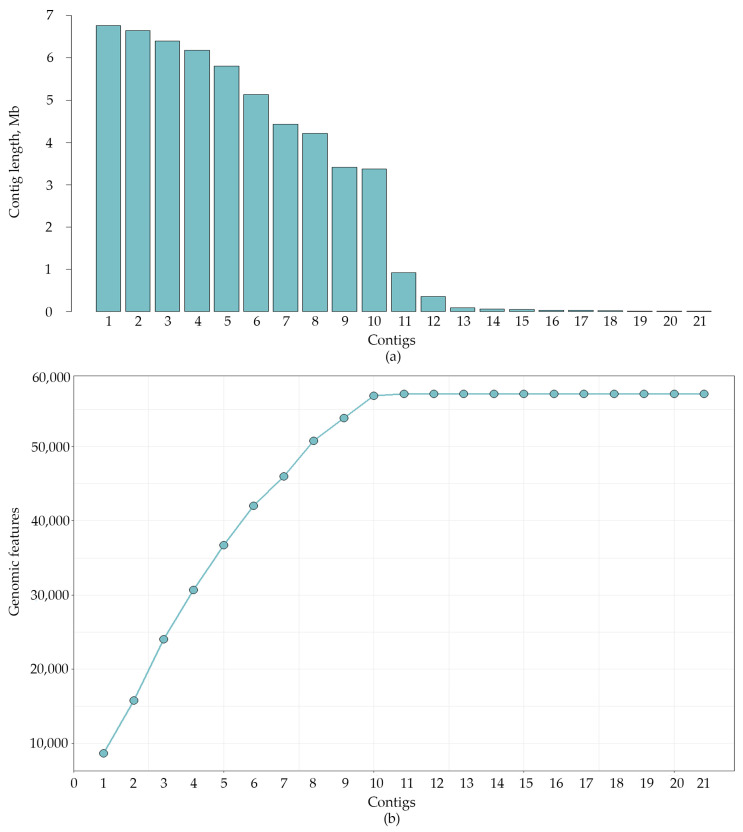
(**a**) Contig length distribution for the *C. lini* strain #394-2 genome assembly. (**b**) The identified complete genomic features for the *C. lini* strain #394-2 genome assembly by the reference-based QUAST analysis. Reference genome—*C. higginsianum* IMI 349063 (NCBI Genome, GCA_001672515.1). Contigs are ordered from the largest (contig 1) to the shortest (contig 21).

The distribution of contig length and the number of genomic features by contigs showed that there were some contigs shorter than 90 kb, which did not contain genomic features (Figure 2).

To analyze the short contigs (#13-21), the *C. lini* strain #394-2 assembly was self-aligned. The results of the self-alignment showed that contigs with a length less than 90 kb consisted of repeats of parts of large contigs and telomeric repeats distributed in a chaotic manner. To make sure that these short contigs can be deleted, we analyzed QUAST and BUSCO statistics of the *C. lini* strain #394-2 assembly without them. According to BUSCO, the number of complete, duplicated, and fragmented genes did not change. The QUAST reference-based parameters, such as covered genome fraction and genomic features, decreased insignificantly from 57.49% to 57.44% and from 57,118 (and 22,393 partial) to 57,116 (and 22,391 partial), respectively. The duplication ratio changed from 1.046 to 1.043. Therefore, we concluded that the short contigs did not have unique information and consisted of repeating information or structural repeats. Thus, the final *C. lini* strain #394-2 genome assembly had 13 contigs, a length of 53.7 Mb, an N50 of 5.8 Mb, and a BUSCO completeness of 96.6%.

### 3.2. Genome Assembly Analyses

**Figure 3:**
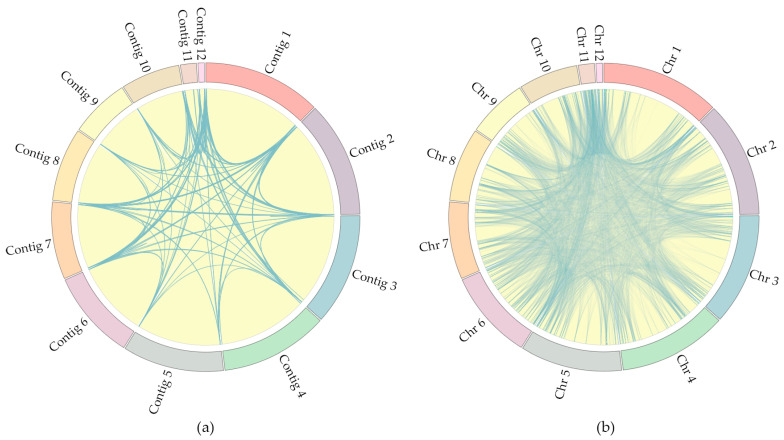
Circos plot of the *C. lini* strain #394-2 genome assembly with links: (**a**) alignments more than 10 kb and more than 96% identity; (**b**) alignments more than 2 kb and more than 96% identity. Grey-blue color lines are links. Contigs and chromosomes goes in the direct order clockwise from the largest to the shortest.

One of the most noticeable quality parameters of the assembly is the presence of telomeric repeats at the ends of the contigs. To identify telomeric repeats in the *C. lini* strain #394-2 genome assembly, we used Tidk and visualized the frequency of telomeric repeat occurrences (Supplementary Figure S1). According to the obtained plots, five contigs had telomeric repeats at one end and seven contigs at both ends. The mitochondrial genome had no telomeric repeats, as expected. Thus, we had seven telomere-to-telomere assembled chromosomes. The telomeric repeats were not found with Tidk at the end of contig 1, contig 3, contig 4, and contig 12, and the start of contig 10. To study these five contigs, we made a self-alignment of the *C. lini* strain #394-2 genome assembly (excluding the mitochondrial genome), considering the alignments of more than 10 kb in length and more than a 96% identity (Figure 3a, Supplementary Figure S2).

According to the Circos plot of such alignments, all contigs had similar ends. Telomeric regions with “TTAGGG” repeats also contained recognizable structures with complex repeats and AT-rich regions. Thus, the ends of the five contigs without telomeric repeats had all these recognizable structures and were linked to the ends of the other contigs with telomeric repeats at both ends. The end of contig 1 was linked with the ends of contig 3 and contig 4 and with the ends of contig 10 and contig 7 and the starts of contig 1, contig 2, and contig 8, which had assembled telomeric repeats. Thus, contig 1, contig 3, and contig 4 were also telomere-to-telomere chromosomes. The start of contig 10 had many links, for example, with the start of contig 9, which had assembled telomeres. The end of contig 12 was linked with its own start and with the start of contig 3. Thus, contig 10 and contig 12 were also telomere-to-telomere chromosomes. Therefore, our assembly represents the complete *C. lini* strain #394-2 genome consisting of 12 linear telomere-to-telomere chromosomes and a circular mitochondrial genome. The contigs were ordered by length from the largest to the shortest and renamed as follows: chromosomes (Chr) from 1 to 12 and the mitochondrial genome.

To understand the structure of the genome, we visualized the links between the chromosomes with a length of more than 2 kb (Figure 3b, Supplementary Figure S3). The number of links suggested the uniqueness of the chromosome sequences. For example, Chr 1 had significantly fewer links than Chr 6. To study the reasons for this observation, we produced the genome annotation.

### 3.3. Genome Assembly Annotation

For the annotation of the *C. lini* strain #394-2 genome assembly, we used RNA-Seq data. We obtained 3.6 million paired Illumina reads (51 + 51 nucleotides). Before annotating, RNA-Seq reads were mapped to the obtained assembly using STAR. The percent of uniquely mapped reads was 93.06%, including 3.05% reads mapped to multiple loci, and 3.89% did not map. RNA-Seq reads covered nearly 20% of the genome, which correlated with the expected approximate number of genes (15 thousand features). The genome assembly was soft-masked with RepeatMasker. Before masking, the assembly contained 0.99 Mb of repeats (1.85% of the genome); 0.88 Mb of masked repeats were simple repeats, and 0.11 Mb were low-complexity regions.

**Figure 4:**
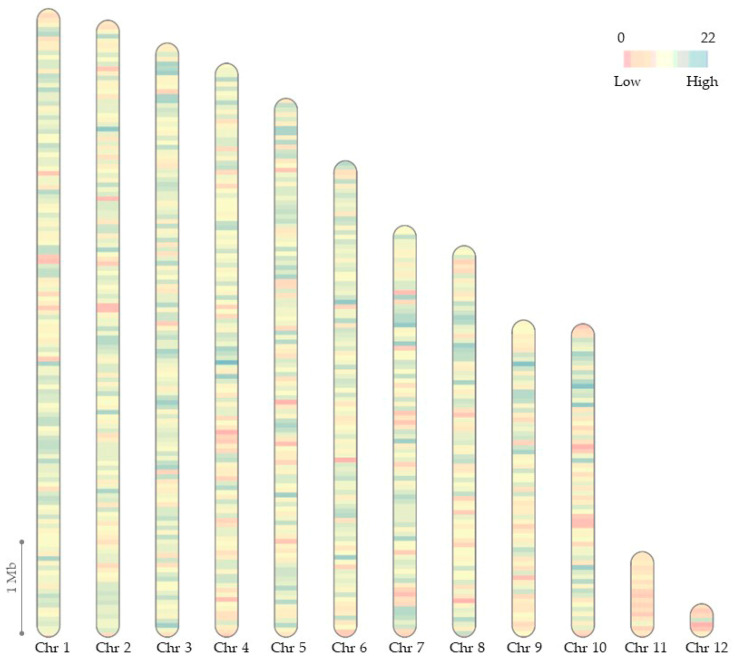
Gene density diagram for 12 chromosomes of the *C. lini* strain #394-2 genome assembly for a 50 kb window. Pink color indicates the lowest gene density (0 genes in 50 kb), blue—the highest gene density (22 genes in 50 kb).

For annotation, we used the Braker tool. To predict genes, Braker uses Augustus and GeneMark. Augustus predicted 12,424 genes, and GeneMark predicted 14,577 genes. The final Braker annotation consisted of 12,449 genes. To observe the distribution of genes by chromosomes, we calculated the gene density for all chromosomes of the *C. lini* strain #394-2 genome assembly in a 50 kb window and visualized it with the R RIdiogram package (Figure 4).

The genome of *C. lini* strain #394-2 consisted of ten core chromosomes and two accessory chromosomes (less than 2 Mb length). The accessory chromosomes had lengths of 0.9 Mb and 0.4 Mb. The core chromosomes had similar gene density distributions. However, Chr 10 had many regions with a high gene density in one half. Accessory chromosomes (Chr 11 and Chr 12) had lower gene densities except for one high gene density region on Chr 12. Chr 11 had no regions with a high gene density at all, and it correlated with the fact that Chr 11 had many links with other chromosomes and might consist of repeats. The accessory chromosomes of fungal pathogens are known to have a lower gene density and a repeat content different from that of core chromosomes [38,39].

**Figure 5:**
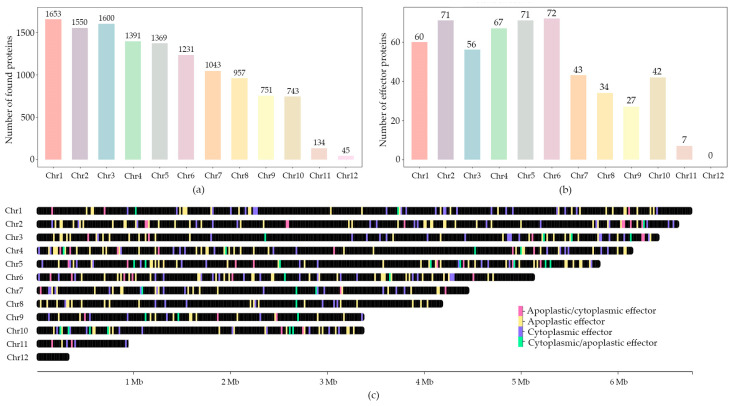
Bar plots illustrating effector gene distribution on chromosomes of the *C. lini* strain #394-2 genome assembly: (**a**) number of identified genes for each chromosome; (**b**) number of identified effector genes for each chromosome; and (**c**) the location of the effector genes on the chromosomes.

The distribution of the genes between the chromosomes is illustrated in Figure 5a. The number of the identified genes correlated with the chromosome size. All the proteins were functionally annotated with InterProScan and also checked for the presence of a signaling peptide with SignalP. Signaling proteins, which accounted for 10% of all identified proteins, were checked for effector features with EffectorP. The program is based on machine-learning techniques and assigns the input proteins to the effector category according to their features. We predicted 550 effector proteins, of which 208 were cytoplasmic effectors and 342 were apoplastic effectors. Chr 6 had the greatest number of effector proteins (Figure 5b). The largest number of effectors relative to chromosome size was in Chr 6 and Chr 10. For understanding the location of the effector genes, we visualized them on the chromosomes (Figure 5c).

The density of effector protein coding genes on the majority of chromosomes was higher at the ends of chromosomes. However, some chromosomes also had many effector proteins in the middle—Chr 6 and Chr 9. Comparing with self-alignment (Figure 3b), the location of the effector proteins on a chromosome was similar with the location of regions aligning to other chromosomes. Therefore, we assumed that the effector protein coding genes could be associated with repeats. Chr 6 had many alignments to the other chromosomes across its whole length, and the predicted effector protein genes were located evenly across the whole length of the chromosome. Chr 1 and Chr 10 had regions near the center of chromosomes with no alignments, and there were less effector proteins in these regions.

### 3.4. Genome Comparison with Chromosome-Level Assemblies of Other Colletotrichum Species

To make a comparison of the obtained complete *C. lini* strain #394-2 genome assembly with the assemblies of other *Colletotrichum* species, we used seven chromosome-level and complete assemblies available in the NCBI Genomes database (https://www.ncbi.nlm.nih.gov/datasets/genome/?taxon=5455&assembly_level=2:3, accessed on 1 July 2024) (Table 1).

**Figure 6:**
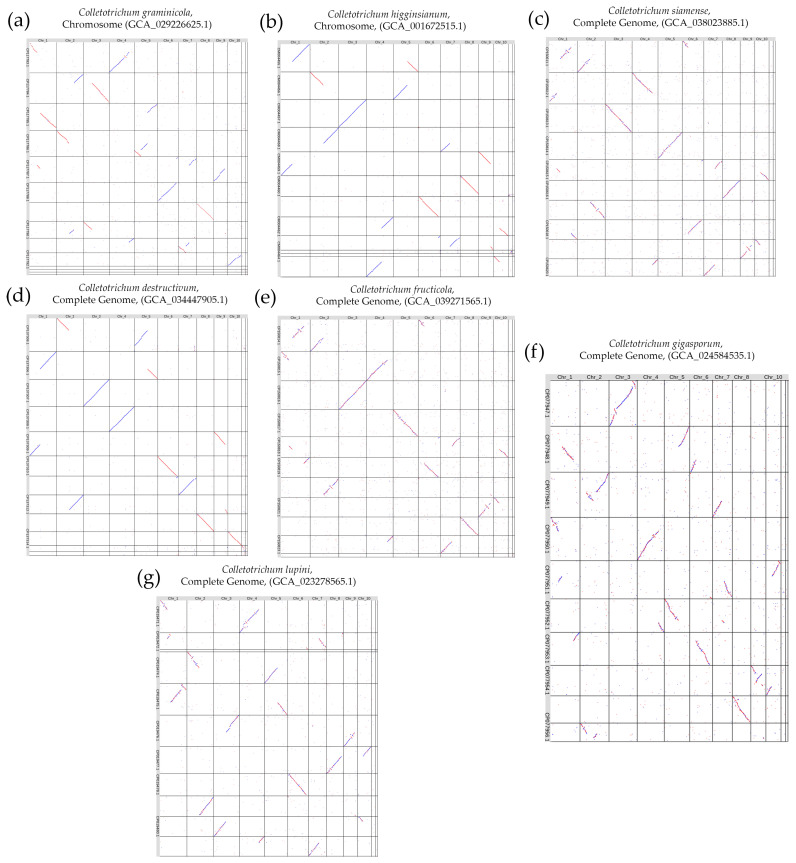
The visualization of the whole-genome LAST alignment of complete and chromosome-level *Colletotrichum* species genome assemblies available in the NCBI Genome database (vertical axis) to the obtained complete *C. lini* strain #394-2 genome assembly (horizontal axis). (**a**) *C. graminicola* chromosome-level genome assembly (GCA_029226625.1); (**b**) *C. higginsianum* chromosome-level genome assembly (GCA_001672515.1); (**c**) *C. siamense* complete genome assembly (GCA_038023885.1); (**d**) *C. destructivum* complete genome assembly (GCA_034447905.1); (**e**) *C. fructicola* complete genome assembly (GCA_039271565.1); (**f**) *C. gigasporum* complete genome assembly (GCA_024584535.1); and (**g**) *C. lupini* complete genome assembly (GCA_023278565.1). The red color indicates direct alignments, the blue color indicates reverse alignments.

The number of chromosomes in the analyzed assemblies of *Colletotrichum* species varied from 10 to 13. We found that the *C. lini* strain #394-2 genome consisted of 12 chromosomes, and this was a characteristic number of chromosomes for this genus. We made whole-genome LAST alignments of the obtained *C. lini* strain #394-2 genome assembly with the seven analyzed assemblies from the NCBI database and visualized them (Figure 6).

According to the alignments, there were many rearrangements between chromosomes of all analyzed *Colletotrichum* species. *C. lini* strain #394-2 Chr 1 aligned to the parts of two to four chromosomes of the other assemblies. Chr 2 had homologous regions with two chromosomes of the analyzed species in most cases. Chr 3 was one of the most conserved ones. This chromosome aligned completely to the chromosomes of *C. higginsianum* (Figure 6b) and *C. destructivum* (Figure 6d), and its full length aligned with rearrangements to the chromosomes of *C. siamense* (Figure 6c), *C. fructicola* (Figure 6e), and *C. gigasporum* (Figure 6f). Chr 4 aligned completely to the chromosome of *C. destructivum* (Figure 6d), while other species had some rearrangements in this chromosome. Other chromosomes of *C. lini* strain #394-2 also showed differences compared to the chromosomes of the other *Colletotrichum* species. For five of the seven analyzed *Colletotrichum* assemblies from the NCBI database, there were from one to three accessory chromosomes. *C. lini* strain #394-2 accessory chromosomes (Chr 11 and Chr 12) did not have extended alignments with chromosomes of the other species. These regions were aligned locally, presented like dots in the alignment visualization. The differences between the chromosomal structures of *C. higginsianum* or *C. destructivum* and *C. lini* strain #394-2 genomes were in the exchange of parts between two chromosomes without other visible rearrangements. Therefore, *C. higginsianum* and *C. destructivum* had the closest chromosome organization with that of *C. lini* strain #394-2.

## 4. Discussion

In this work, we obtained the complete annotated genome assembly of the highly virulent *C. lini* strain. The genome was covered with long ONT reads ~45 times and with short Illumina reads ~90 times (an estimated genome size of 54 Mb). The draft assembly was obtained using the Canu assembler and polished with Racon ×2 and Medaka using ONT reads and Polca using Illumina reads. This assembly scheme showed the greatest efficiency for this species in our previous study [10], and it was effective in the present work, too.

Next, to clear the assembly of repetitive contigs, self-alignment and searches for the mitochondrial genome were performed. It turned out that the assembly contained five contigs with several copies of the mitochondrial genome. We left one complete copy; the rest of the repetitions were deleted from the assembly. Further, considering the results of the self-alignment, we found that some parts of several short contigs were similar to the repeating parts of large contigs, and the other parts of these contigs were chaotically similar to various telomeric repeats. Such contigs were also removed from the assembly. To prove our judgments, we compared the BUSCO completeness, as well as the reference-based QUAST statistics of the assemblies before and after filtering the contigs. The completeness of the assembly did not decrease, as demonstrated by any of the parameters. This suggested that these contigs were indeed the result of an imperfect assembly.

Thus, the final genome assembly consisted of thirteen contigs, one of which was the mitochondrial genome. The obtained assembly length was 53.7 Mb with N50 of 5.8 Mb. The assembly BUSCO completeness was 96.6%. According to the results of the comparison between the obtained *C. lini* genome assembly and the available chromosome-level assemblies of *Colletotrichum* species, 10 core chromosomes is a common number of chromosomes for the genus *Colletotrichum*. For example, *C. destructivum* and *C. higginsianum* have ten core and two accessory chromosomes with a genome size of 51–52 Mb [40] (https://www.ncbi.nlm.nih.gov/datasets/genome/GCF_001672515.1/, accessed on 1 July 2024). Five of the seven complete and chromosome-level assemblies available in the NCBI database also have accessory chromosomes. Their number ranges from one to three. Two assemblies (*C. gigasporum* and *C. siamense*) do not contain accessory chromosomes.

When comparing the genome structures of different *Colletotrichum* species, we aligned the resulting *C. lini* genome with the available chromosome-level assemblies from the NCBI database. It turned out that *C. higginsianum* and *C. destructivum* were the species closest by genome structure to *C. lini*. Rearrangements between the chromosomes, numbering from two to three per chromosome, were most common in these genomes. Species with different host plants had different rearrangements of the core chromosomes. Studies devoted to this described this phenomenon in terms of chromosomal rearrangements (CRs) [41].

After searching for telomeric repeats, we revealed that seven *C. lini* chromosomes were assembled completely from telomere to telomere, and the remaining five contigs had telomeric repeats at one end. After visualizing the results of *C. lini* genome self-alignments of a length of more than 10 kb and an identity of more than 96%, we found that areas with assembled telomeres were aligned to the ends of the contigs, which did not have completely assembled telomeric repeats. Thus, all chromosomes were completely assembled, although some of them did not have telomeric repeats. We assume that the short contigs, which contained chaotically distributed telomeric repeats and were removed during the assembly improvement, are the missing telomeres. This could be explained by the fact that assembling telomeric repeats, as any other repeats, is a difficult task for assembly algorithms [42]. Moreover, the self-alignment showed that repeats at the ends of chromosomes are the longest in the *C. lini* genome. To try to solve such issues, several tools have been developed, e.g., Verkko, quarTeT, and Chrom-pro [43,44,45]. Finding and assembling telomeric repeats depends not only on the assembler algorithm, but also on the location of reads spanning the telomeres, which happens randomly when DNA is isolated. The basecalling algorithms for ONT reads also have problems with telomeric repeats [46]. However, telomeres are characterized not only by telomeric repeats themselves, but also by recognizable repeats and structures before them. These repeats and structures can be detected by self-aligning the genome and making sure that the chromosome is assembled from telomere to telomere.

In addition, to understand the structure of the chromosomes, the results of the self-alignment with a length of more than 2 kb were visualized. It turned out that some chromosomes had significantly more unique sites than others.

Using RNA-Seq data, we annotated 12,449 genes. This number was in line with the number of genes for other chromosome-level assemblies of *Colletotrichum* species [15,47]. The available annotated complete and chromosome-level assemblies had 14,651–18,742 predicted proteins, and this number correlated with the genome size. We tested the obtained protein sequences for the presence of a signaling peptide, as well as for effector functions, and identified 550 effector proteins. On predicting the secretion signal in the studied protein set, we used EffectorP, which makes a general prediction on effector functions. However, the potential host targets and the role of the classified proteins should be studied and confirmed experimentally. After classification, we studied the location and distribution of effector genes across the chromosomes. One of the studies on *C. fructicola*, *C. siamense*, *C. aenigma*, *C. tropicale*, and *C. viniferum* revealed that clusters of effector genes on accessory chromosomes are associated with telomeres and repeat-rich chromosomes [16]. According to our results, the same trend exists for both accessory and core chromosomes of *C. lini*. Paying attention to the distribution of genes along chromosomes, it can be seen that Chr 6, which has repeats throughout its entire length, also has effector genes along its entire length. Meanwhile, Chr 4, Chr 10, and Chr 11 have a distribution of effector genes similar to that found in the repetitive regions and areas close to telomeres.

The first complete annotated *C. lini* genome assembly obtained in this study opens up huge opportunities for further *C. lini* genome research, including the determination of pathogenicity mechanisms, and for the comparative analysis of strains within the species and within the entire genus *Colletotrichum*.

## Figures and Tables

**Table 1:** Complete and chromosome-level genome assemblies of *Colletotrichum* species available in NCBI, which were used for the comparison with the obtained complete *C. lini* strain #394-2 genome assembly.

Species	Assembly Level	Number of Chromosomes (Core + Accessory)	Genome Size, Mb	Contig N50, Mb	Gene Count	Assembly Accession
*C. lini*	Complete	12 (10 + 2)	53.7	5.8	12,449	-
*C. graminicola*	Chromosome	13 (10 + 3)	57.4	5.0	15,185	GCA_029226625.1
*C. higginsianum*	Chromosome	12 (10 + 2)	50.7	5.2	14,651	GCA_001672515.1
*C. destructivum*	Complete	12 (11 +1)	51.8	5.3	15,631	GCA_034447905.1
*C. fructicola*	Complete	11 (10 + 1)	56.4	4.9	-	GCA_039271565.1
*C. gigasporum*	Complete	10	87.0	9.2	-	GCA_024584535.1
*C. lupini*	Complete	11(10 + 1)	63.4	7.9	18,742	GCA_023278565.1
*C. siamense*	Complete	10	56.8	6.7	-	GCA_038023885.1

## Data Availability

The generated dataset for this study can be found in the NCBI database under the BioProject accession number PRJNA929545.

## Supplementary Materials

The following supporting information can be downloaded at: https://www.mdpi.com/article/10.3390/jof10090605/s1, Figure S1: Telomeric repeat (TTAGGG) content along the contig sequences of *C. lini* strain #394-2; Figure S2: Circos plot of the *C. lini* strain #394-2 genome with links of length more than 10 kb and more than 96% identity; Figure S3: Circos plot of the *C. lini* strain #394-2 genome with links of length more than 2 kb and more than 96% identity; Table S1: QUAST statistics of the *C. lini* strain #394-2 genome assembly at each step.
