# Supplementary figures and images for: Complete Annotated Genome Assembly of Flax Pathogen Colletotrichum lini
Source: J Fungi (Basel). 2024 Aug 26;10(9):605. doi: 10.3390/jof10090605 (PMC11432806; doi:10.3390/jof10090605)

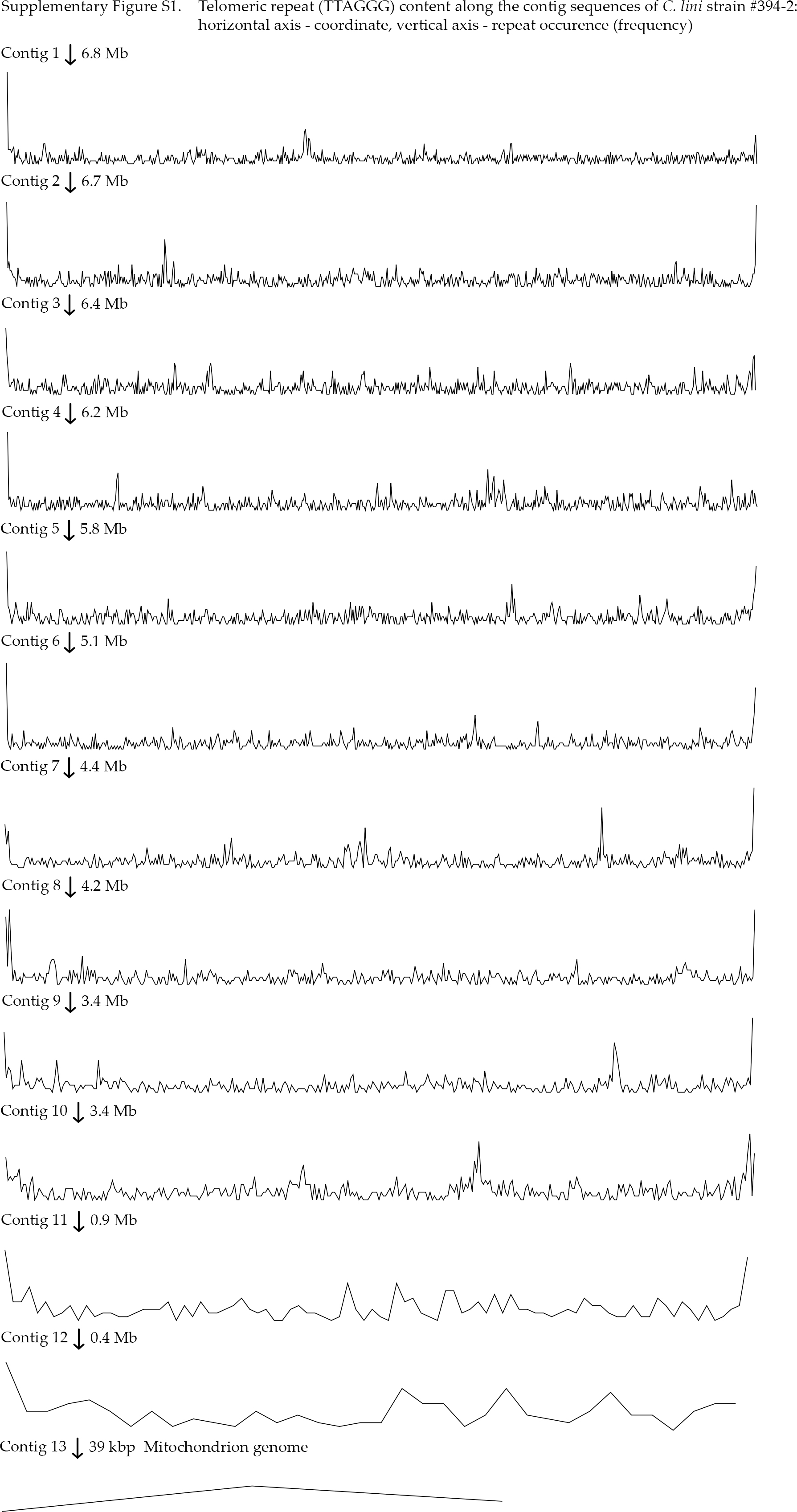

Supplement: Supplementary file 1 [file jof-10-00605-s001.zip › Supplementary_Figure_S1.jpg]
